# Expression and Clinical Implications of Cysteine Cathepsins in Gallbladder Carcinoma

**DOI:** 10.3389/fonc.2019.01239

**Published:** 2019-11-22

**Authors:** Siddharth Mehra, Rajesh Panwar, Bhaskar Thakur, Rajni Yadav, Manish Kumar, Ratnakar Singh, Nihar Ranjan Dash, Peush Sahni, Shyam S. Chauhan

**Affiliations:** ^1^Department of Biochemistry, All India Institute of Medical Sciences, New Delhi, India; ^2^Department of G.I. Surgery and Liver Transplantation, All India Institute of Medical Sciences, New Delhi, India; ^3^Department of Biostatistics, All India Institute of Medical Sciences, New Delhi, India; ^4^Department of Pathology, All India Institute of Medical Sciences, New Delhi, India

**Keywords:** gallbladder carcinoma, cathepsin L, cathepsin B, enzyme activity, expression, serum biomarker

## Abstract

**Background:** Gallbladder carcinoma (GBC) exhibits poor prognosis due to its detection at an advanced stage. Upregulation of lysosomal cysteine proteases cathepsin L (CTSL) and cathepsin B (CTSB) has been implicated in several tumorigenic processes. However, no such information in GBC was available. Therefore, the present study was planned to investigate the expression and clinical significance of these cathepsins in GBC.

**Methods:** Activities of CTSL and CTSB were assayed in the gallbladder (GB) tissues obtained from GBC patients (*n* = 43) and control subjects (*n* = 69). Protein and mRNA levels were quantified using immunohistochemistry and real-time PCR (qPCR), respectively. Finally, serum levels of CTSL and CTSB were estimated by ELISA. Receiver operating characteristic (ROC) curve analysis was used for the assessment of sensitivity, specificity, and diagnostic accuracy of these cysteine cathepsins in GBC. The association of combined CTSL and CTSB activity with overall survival was assessed using Kaplan Meier survival analysis.

**Results:** The expression and activity of both CTSL and CTSB were significantly increased (*p* < 0.050) in tumors of GBC patients as compared to controls. Enzymatic activity of CTSL+B and CTSB exhibited a strong positive association with tumor stage and lymph node involvement in GBC (*p* < 0.050). Interestingly, the elevated activity of combined CTSL+B was also associated with increased mortality in these patients. Furthermore, significantly enhanced levels of serum CTSL and CTSB were also observed in GBC (*p* < 0.050) as compared to controls. ROC analysis revealed high diagnostic significance of serum CTSB and CTSL for distinguishing GBC patients from controls with an area under the curve (AUC) of 82 and 77%, respectively.

**Conclusion:** This study, for the first time, demonstrates the clinical significance of CTSL and CTSB overexpression in GBC. Our findings may help improve the clinical management of this carcinoma.

## Introduction

Gallbladder carcinoma (GBC) is an aggressive neoplasm, accounting for 80–90% of all bile tract malignancies (1). It is the fourth most common cancer among females in North India (2), with a 1-year survival rate of only 10% for advanced-stage GBC (3). Curative radical resection is considered the mainstay treatment of GBC. However, due to the lack of early symptoms and specific biomarkers, most patients present with an advanced stage of the disease, precluding them from the surgery (4). Therefore, there is an urgent need to identify novel biomarkers for early detection.

Carcinoma of the gallbladder is associated with the worst prognosis mainly because of the high incidence of micrometastases into adjacent sites, including liver parenchyma, bile duct, blood vessels, and regional lymph nodes (5). This critical process of tumor invasion into the surrounding tissue requires several modifications in the extracellular matrix (ECM) and is aided by the active participation of several proteases (6). Cysteine cathepsins CTSL and CTSB belong to the papain subfamily of ubiquitous lysosomal proteases and are responsible for normal turnover and degradation of intracellular proteins (7–9). Altered localization and increased expression of these cysteine cathepsins have been implicated in invasion and metastasis of numerous other malignancies (10–12). After being released by tumor cells, these proteolytic enzymes break down components of the ECM such as collagen, laminin and elastin, thereby allowing for the dissemination of primary tumor cells (13). Diagnostic utility of these cathepsins in serum samples of colorectal and pancreatic cancer patients has also been established previously (11, 14). Using quantitative proteomics analysis, Shahasrabuddhe et al. observed overexpression of cathepsin H and Z in gallbladder cancer (15). However, no information about the expression or significance of CTSL and CTSB in the pathogenesis of GBC was available.

Thus, the present study aimed to examine the expression and clinical relevance of cysteine cathepsins (CTSL and CTSB) in both tissue and serum of GBC patients.

## Materials and Methods

### Patient Selection Criteria and Samples

Ethical clearance from the Human Ethics Committee of All India Institute of Medical Sciences was obtained before commencement of the study (IESC T-244/2012), and informed consent from all patients or their legally acceptable representatives was taken before their inclusion. All patients included in this study underwent treatment at the Department of Gastrointestinal Surgery (GIS) at the All India Institute of Medical Sciences (AIIMS), New Delhi.

In the present study, surgically resected gallbladder tumor tissues were obtained from GBC patients (*n* = 43) who underwent a presumed curative surgical resection. All cases were staged clinically, according to the American Joint Committee on Cancer (AJCC) guidelines. Tissue samples obtained from patients undergoing cholecystectomy for gallstone disease and from patients with periampullary carcinoma where the normal gallbladder was removed as a part of pancreaticoduodenectomy were also collected and served as controls (Total Controls, *n* = 69). These gallbladders were histologically proven chronic cholecystitis with no evidence of any malignancy. A section of all resected gallbladder tissues (both tumor and controls) was immediately snap-frozen in liquid nitrogen and stored at −80°C to be used for enzymatic assays and RNA isolation. Another portion of the resected tissue was fixed in 10% neutral buffered formalin solution for immunohistochemical analysis.

**Figure 1:**
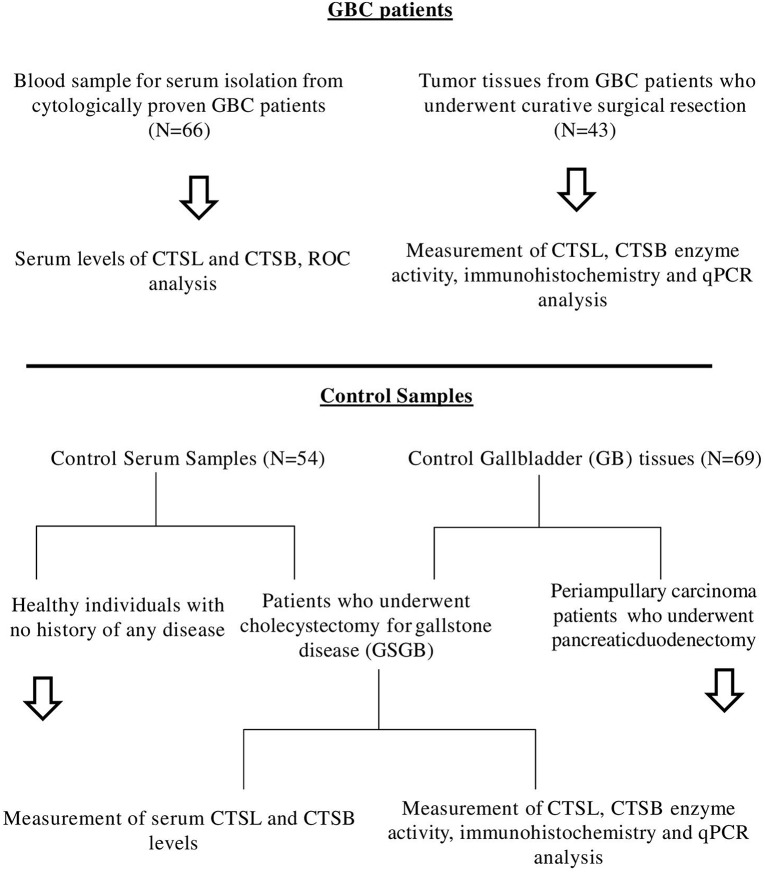
Schematic representation of sample collection and work flow for GBC patients and controls used in this study.

Further, preoperative serum samples were obtained from cytologically proven cases of GBC (*n* = 66, median age 54 years, males = 23 and females = 43) including both resectable and locally advanced/metastatic GBC, attending the Outpatient Department of GIS. For controls, cholecystitis patients with gallstone gallbladder disease (GSGB) who underwent cholecystectomy (*n* = 34) and healthy individuals (*n* = 20) with no active inflammation, gallstones, or malignancy were recruited in the present clinical setup. All blood samples were processed for serum isolation and stored at −80°C until used for further analysis. The schematic representation of sample collection and workflow for GBC patients and controls is outlined in Figure 1.

### Enzyme Assay for CTSL and CTSB in Gallbladder Tissues

A total of 5–10 mg of frozen gallbladder tissue was lysed in Tris-HCl buffer (50 mM Tris-HCl, pH6.8; 150 mM NaCl; 10% Glycerol; 1% Nonidet P-40). After two cycles of freeze-thaw, the homogenate was centrifuged at 10,000 × g at 4°C for 15 min to remove cell debris. Subsequently, total protein in the supernatant was estimated by BCA protein estimation. An equal amount of 100 μg protein was used to assay the combined CTSL+B activity in the supernatant using CBZ-Phe-Arg-NMec (Sigma-Aldrich, U.S.A), a synthetic fluorogenic substrate. Simultaneously, the same assay was performed in the presence of 5 μM CA074 Me (Calbiochem, Germany), a specific CTSB inhibitor to measure CTSL activity. Values obtained from CTSL activity were subtracted from total CTSL+B activity to calculate CTSB enzyme activity. The enzymatic activities were expressed as Relative Fluorescence Units (RFU)/min/mg protein.

### Immunohistochemical Analysis of CTSL and CTSB

Immunohistochemistry was performed on 4 μm thick paraffin-embedded tissue sections of control and carcinoma gallbladder using mouse monoclonal anti CTSL (1:500, ab6314, Abcam, USA) and CTSB antibody (1:200, ab58802, Abcam, USA) as described previously (16). Briefly tissue sections were mounted on glass slides and deparaffinized in xylene, xylene: alcohol, and alcohol gradients, followed by antigen retrieval in citrate buffer (0.01 M, pH 6:0) using a microwave oven. The slides were allowed to cool at room temperature and incubated with 3% serum for 30 min to preclude non-specific binding. These sections were then incubated with the primary antibody in a humidified chamber at 4°C overnight. On the following day, slides were washed thrice with Tris buffer saline (TBS), and sections were treated with hydrogen peroxide (0.3% v/v in ethanol) for 20 min to quench the endogenous peroxidase activity. Primary antibody was detected using VECTASTAIN® Elite® ABC peroxidase kit as per the manufacturer's protocol using diaminobenzidine (DAB) as the chromogen. Finally, the sections were counterstained with Mayer's hematoxylin and mounted with D.P.X. Tissue sections were microscopically examined.

Two pathologists independently performed immunohistochemistry scoring. A semi-quantitative H-score was used for evaluating CTSL and CTSB immunostaining pattern. It was calculated by the addition of staining intensity and percentage distribution scores evaluated in epithelial cells, tumor cells, and endothelial cells. The staining intensity was scored from 0 to 3 as follows: 0 (no staining), 1 (weak), 2 (moderate), and 3 (strong). Percentage distribution was scored as follows: No staining = 0, < 40% = 1, 40–80% = 2, > 80% = 3. Thus, the H-score ranged from 0 to 6. Based on this score, immunoexpression of CTSL and CTSB was divided into two subgroups: Low (H score, 1–4) and high (H score 5–6) expression groups.

### RNA Isolation and qPCR in Gallbladder Tissues

Total cellular RNA was extracted from snapped frozen gallbladder tissues using TRI Reagent BD™ (Sigma-Aldrich, U.S.A) as per the manufacturer's instructions from snapped frozen tissues. The procedure followed for cDNA preparation, and qPCR has been described previously (17). Transcripts were normalized using 18S ribosomal RNA. After normalization, 2^−ΔCt^ values obtained were plotted in a dot plot. The nucleotide sequences of primer sets used for human CTSL, CTSB, and 18S are given in Supplementary Table 1.

### Enzyme-Linked Immunosorbent Assay in Serum (ELISA)

Levels of serum CTSL and CTSB were assayed using a commercially available sandwich ELISA kits (RayBiotech, Norcross, GA and Bosterbio, Pleasanton, CA, U.S.A.) as per the manufacturer's instructions as described in detail elsewhere (18).

### Statistical Analysis

STATA 13.1 software (Lakeway Drive College Station, Texas USA) was used for all statistical evaluation. The skewed distributed variables were analyzed by the Mann-Whitney *U* test and were presented as median (interquartile range, IQR). The cut-off values of CTSL and CTSB levels (in tissues and serum) were determined using receiver operating characteristic (ROC) curves analysis along with their associated diagnostic measures. Chi-square test assessed the association between two categorical variables in semi-quantitative immunohistochemistry scoring. Of the 43 patients who were operated, only 35 underwent curative surgery (Radical cholecystectomy); as a result, these 35 patients were enrolled in the follow-up protocol to assess the association of CTSL and CTSB over expression with survival after presumed curative resection. The additional 8 patients could not undergo a radical cholecystectomy in view of metastatic or locally advanced unresectable disease that was detected during surgery. The overall survival time of the patients (in months) was calculated from the date of their surgery to the date of the last follow-up. To verify whether CTSL+B, CTSL, or CTSB expression correlated with survival, these variables were dichotomized according to their median values, and Kaplan–Meier analysis was performed using log-rank tests. Spearman rank correlation coefficient (ρ) analysis was used to assess the correlations between the activity and mRNA levels of cysteine cathepsins. All tests were two-sided, and ^*^p ≤ 0.05, ^**^p ≤ 0.01, and ^***^p ≤ 0.001 were considered to be statistically significant.

## Results

### Characteristics of GBC Patients

All subjects enrolled in the present study were ethnic South Asians residing in the northern part of India. The median age group of GBC patients who underwent curative surgical resection in the study group was 56 years. Out of 43 patients, 11 were males and 32 females. Based on the TNM stage classification, 17 GBC patients were stage I ± II and 26 were stage III ± IV. The other details including tumor size, histological type, grading, the involvement of lymph node and liver in these patients are given in Table 1.

**Table 1:** Association of clinicopathological variables with CTSL+B, CTSL, and CTSB enzymatic activity in GBC tissues.

**Clinical Parameters**	**Number of Cases**	**CTSL+B Activity (RFU/min/mg protein)**	***p* value**	**CTSL Activity (RFU/min/mg protein)**	***p* value**	**CTSB Activity (RFU/min/mg protein)**	***p* value**
**AGE**							
≤56 years	23	127.7 (55.4–184.6)	0.443	8.3 (2.5–14.1)	0.128	102.3 (50.0–179.0)	0.550
> 56 years	20	106.4 (43.3–142.5)		5.1 (1.8–7.1)		95.5 (40.9–136.2)	
**SEX**							
Males	11	109.6 (55.4–152.8)	0.823	6.6 (3.6–17.4)	0.303	94.3 (50–139.9)	0.616
Females	32	108.9 (46.7–199.4)		5.8 (2.0–11.3)		102.0 (43.8–193.6)	
**HISTOLOGICAL TYPE**							
Adenocarcinoma	37	110.4 (53.2–181.5)	0.592	5.6 (2.5–11.4)	0.740	102.3 (48.61–174.5)	0.307
Others (Adenosquamous)	6	105.9 (24.2–166.2)		9.6 (0.9–30.0)		81.5 (23.3–130.2)	
**HISTOLOGICAL GRADING**							
Well/ moderate	33	109.6 (50.9–178.4)	0.423	5.6 (2.4–12.9)	0.581	95.7 (47.2–170.0)	0.361
Poor	7	153.5 (68.4–332.9)		6.6 (3.6–11.4)		147.1 (66.4–321.5)	
**TUMOR SIZE**							
≤4 cm	22	104.8 (33.4–178.4)	0.281	5.8 (1.7–12.9)	0.487	90.7 (31.1–170)	0.202
> 4 cm	17	125.6 (68.4–184.6)		6.3 (5.2–11.4)		104.2 (66.4–179)	
**T STAGE**							
T1+T2	15	55.4 (14.9–152.8)	**0.028**	4.5 (1.4–7.8)	0.065	50.0 (13.4–139.9)	**0.028**
T3+T4	26	126.6 (100.9–184.6)		6.9 (3.7–14.1)		114.8 (70.7–179.0)	
**TNM STAGING**							
I+II	17	102.2 (33.4–138.6)	0.117	6.0 (1.7–14.1)	0.746	87.2 (31.1–121.6)	0.073
III+IV	26	118.0 (68.4–234.2)		5.8 (3.6–11.3)		106.1 (63.1–229.6)	
**LYMPH NODE INVOLVEMENT**							
No	30	106.5 (33.4–152.8)	**0.045**	5.6 (2.2–12.9)	0.325	96.6 (31.1–139.9)	**0.045**
Yes	10	189.9 (100.9–249.0)		8.9 (5.2–11.4)		178.0 (92.2–237.7)	
**LIVER INVOLVEMENT**							
No	32	106.5 (34.5–165.9)	0.188	5.8 (2.1–10.9)	0.183	93.2 (32.8–158.6)	0.114
Yes	10	126.6 (100.9–249.0)		10.8 (4.2–19.6)		116.2 (95.7–237.7)	

Gallbladder tissues obtained from all subjects were processed for preparation of lysate and assay of enzymatic activities as described in the Material and Methods section. Enzymatic activity values have been expressed as median (IQR) ×10^3^ RFU/min/mg protein. Results were statistically analyzed by the Mann-Whitney U test and p values < 0.050 were considered to be significant. CTSL, cathepsin L; CTSB, cathepsin B; RFU, relative fluorescence units; GBC, gallbladder cancer.

*Bold values indicate p < 0.050*.

### Enzyme Activity of CTSL and CTSB in GBC

**Figure 2:**
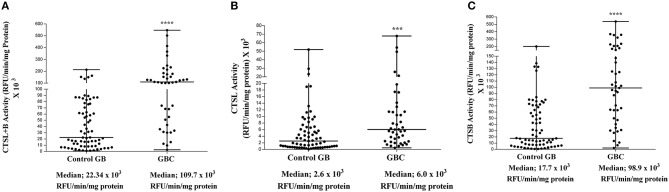
Enzyme activity of cysteine cathepsins in GBC. Activity of each cathepsin in tissue samples of control gallbladder (*n* = 69) and GBC (*n* = 43) was assayed spectrofluorometrically as described in the Materials and Methods section. The activity of **(A)** CTSL+B, **(B)** CTSL, and **(C)** CTSB in each subject has been plotted in a dot plot. The median value in each case has been represented by a horizontal bar in the middle. Enzymatic activities of both the cathepsins were significantly higher in GBC than that of controls. All values presented as median with interquartile range (IQR). Results were analyzed using the Mann Whitney *U* test. ***p ≤ 0.001 and ****p ≤ 0.0001 were considered statistically significant.

The enzymatic activity of CTSL and CTSB was assayed in tumor and control gallbladder tissues using a synthetic fluorogenic substrate CBZ-PheArg-NMec and expressed as RFU/min/mg protein. The median CTSL+B activity in GBC patients was 109.6 × 10^3^ RFU/min/mg protein (IQR 44.6–177.4) as compared to 22.3 × 10^3^ RFU/min/mg protein (IQR 8.2–73.1) in control gallbladder. Thus, the median CTSL+B activity was significantly higher (5-fold) in GBC with respect to controls (*p* < 0.0001, Figure 2A). Similarly, the median CTSL activity in the same set of patients was 6.0 × 10^3^ RFU/min/mg protein (IQR 2.4–11.4), ~2.4-fold more than that in controls (2.6 × 10^3^ RFU/min/mg protein, IQR 0.7–6.6, *p* = 0.0004, Figure 2B). Interestingly, as compared to controls (17.7 × 10^3^ RFU/min/mg protein, IQR 7.1–65.7,) a drastic increase of around 5-fold in CTSB activity was also observed in GBC patients (98.9 × 10^3^ RFU/min/mg protein; IQR 47.2–170.1, *p* < 0.0001, Figure 2C). Furthermore, we observed a strong correlation between the activities of these proteases and clinicopathological parameters of GBC patients. The elevated activity of CTSL+B, as well as CTSB, displayed a significant association with the T stage and lymph node involvement (Table 1, *p* < 0.050).

### Diagnostic and Prognostic Significance of Cysteine Cathepsins in GBC

**Figure 3:**
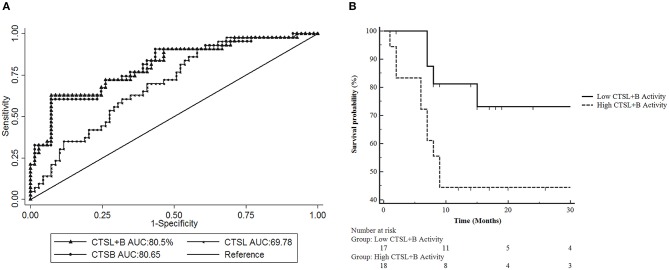
Receiver Operative Characteristic (ROC) Curve analysis of CTSL+B, CTSL, and CTSB in GBC. **(A)** ROC curve analysis revealed excellent diagnostic performance of CTSL+B and CTSB with an AUC value of 80% in GBC. Sensitivity = True positive fraction; 1-Specificity = False-positive fraction. **(B)** Prognostic significance of cysteine cathepsins in GBC. Kaplan Meier analysis revealed a strong correlation between higher CTSL+B activity and poor overall survival in GBC patients (*p* = 0.0493).

Having established increased activities of both CTSL and CTSB in GBC as compared to control gallbladder with chronic cholecystitis, we then sought to examine the diagnostic potential of these cathepsins in GBC using ROC analysis (Figure 3A and Table 2).

**Table 2:** Diagnostic performance assessment for cysteine cathepsins in GBC tissues.

**Cysteine cathepsins** **(Cut off values)**	**AUC** **(%, 95% CI)**	**Sensitivity** **(%, 95% CI)**	**Specificity** **(%, 95% CI)**	**PPV** **(%, 95% CI)**	**NPV** **(%, 95% CI)**
CTSL+B (68 × 10^3^ RFU/min/mg)	80 (71–88)	72 (56–84)	74 (61–83)	63 (48–76)	80 (68–89)
CTSB (63 × 10^3^ RFU/min/mg)	80 (72–89)	72 (56–84)	74 (61–83)	63 (48–76)	80 (68–89)
CTSL (4.5 × 10^3^ RFU/min/mg)	69 (60–79)	62 (46–76)	65 (52–76)	52 (38–66)	73 (60–83)

*AUC, area under the curve; NPV, negative predictive value; PPV, positive predictive value. AUC was calculated using receiver–operating characteristic curves. Cut off values were determined by maximizing the sum of sensitivity and specificity*.

Our analysis revealed an optimal cut-off value of 68 × 10^3^ RFU/min/mg protein for CTSL+B in GBC with 72% diagnostic sensitivity and an AUC value of 80%. Thus, 72% of GBC patients (31/43) displayed higher activity of CTSL+B than the assigned cut-off value. For CTSL, the identified cut off was 4.5 × 10^3^ RFU/min/mg protein, with 62% diagnostic sensitivity and an AUC value of 69%. This again translated to a reasonable diagnostic accuracy with 62% (27/43) of GBC patients having higher values than the calculated cutoff for CTSL. Similarly, the identified cut-off value for CTSB was 63.1 × 10^3^ RFU/min/mg protein, with 72% sensitivity and an AUC value of 80%, again distinguishing over 72% of GBC patients with increased CTSB activity as compared to only 28% (12/43) of cases with values lower than the cut-off. These results imply the excellent diagnostic performance of combined CTSL+B and CTSB activity values in differentiating GBC from chronic cholecystitis in gallbladder tissues.

Further, we aimed to determine if the higher activity of these cathepsins also correlates with poor survival in GBC patients who underwent curative surgical resection (*n* = 35). For this, the median CTSL+B activity values were taken as a cut-off to divide GBC patients into two subgroups, i.e., one with the higher median enzymatic activity and the other with the lower values than the cut-off. Kaplan-Meier analysis revealed significantly reduced overall survival in patients with higher CTSL+B activity (median value >107.5 × 10^3^ RFU/min/mg protein) as compared to patients with lower values (*p* = 0.049, log-rank test, hazard ratio 2.93, 95% CI = 1.02–8.39, Figure 3B). These results indicate that a combined increase in the activities of CTSL+B not only correlates with the active disease but also portends a poor prognosis in GBC.

### Immunohistochemical Analysis of CTSL and CTSB in GBC

**Figure 4:**
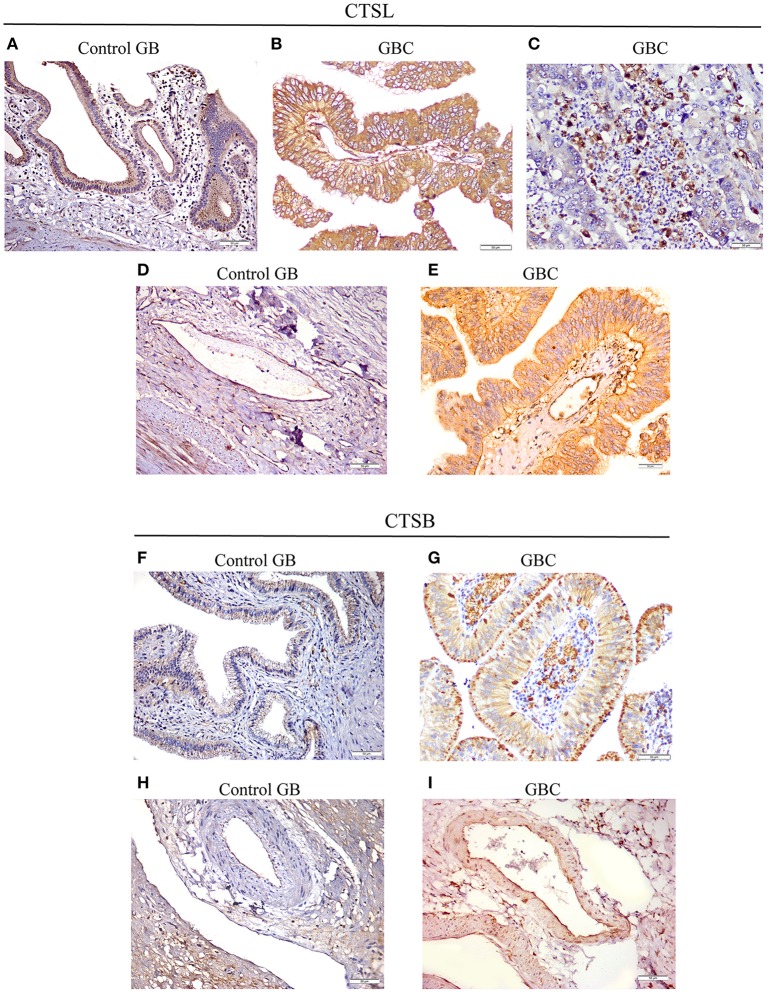
Immunohistochemical analysis of CTSL and CTSB in GBC. **(A)** Control gallbladder (GB) tissue exhibiting faint immunopositivity for CTSL in epithelial cells of normal mucosa and **(D)** endothelial cells, while in GBC tissues prominent expression of CTSL was noted in **(B)** tumor cells, **(C)** tumor-associated macrophages and **(E)** tumor endothelial cells. Similarly, for CTSB, weak cytoplasmic positivity was observed in **(F)** normal mucosa and **(H)** endothelial cells of controls, whereas increased expression of CTSB was noted in the cytoplasmic compartment of **(G)** tumor cells, tumor-associated macrophages and **(I)** tumor endothelial cells of GBC tissues (Original magnification ×200, *scale bar* 50 μm).

Next, we sought to study the protein expression and localization of CTSL and CTSB in gallbladder tissues. For this immunohistochemical analysis was performed on paraffin-embedded tissue sections of gallbladder obtained from GBC patients and controls. A weak cytoplasmic immunopositivity was noted for both CTSL and CTSB at the apical surface of the columnar epithelium and endothelial cells of control gallbladder. However, staining for both these proteases was very strong in tumor cell populations, tumor-associated macrophages, and tumor endothelial cells of histologically proven GBC tissues (Figures 4A–I). Among total cases of GBC, 77% (31/40) displayed increased expression of CTSL (H-score>4) in tumor cells as compared to only 16% (10/60) of control gallbladder tissues (Figures 4A,B). Similarly, intense staining of CTSL in tumor endothelial cells was also noted in 55% (22/40) of GBC patients as compared to only 20% (12/60) in endothelial cells of controls (Figures 4D,E), indicating a remarkable increase in CTSL levels in GBC (*p* < 0.0001, Table 3). Interestingly, immunohistochemical staining of CTSB was markedly higher (H-score >4) in 67% (27/40) of the GBC patients compared to only 10% (6/60) in control gallbladder sections (Figures 4F,G). Likewise, 75% (30/40) of GBC cases also exhibited higher CTSB expression (H-score >4) in tumor endothelial cells as compared to only 11% (7/60) of controls, implying significant overexpression of CTSB protein (*p* < 0.0001, Figures 4H,I, Table 3) in this malignancy.

**Table 3:** Assessment of cysteine cathepsin expression in gallbladder tissues by immunohistochemical analysis.

	**Control GB**			**GBC**			
**H Score**		**High expression** ***N* (%)** **H score > 4**	**Low expression** **N (%)** **H score ≤ 4**		**High Expression** **N (%)** **H score > 4**	**Low Expression** **N (%)** **H score ≤ 4**	***p* value**
CTSL	Epithelial cells	10/60 (16%)	50/60 (83%)	Tumor cells	31/40 (77%)	9/40 (22%)	<0.0001
	Endothelial cells	12/60 (20%)	48/60 (80%)	Tumor endothelial cells	22/40 (55%)	18/40 (45%)	<0.0001
CTSB	Epithelial cells	6/60 (10%)	54/60 (90%)	Tumor cells	27/40 (67%)	13/40 (32%)	<0.0001
	Endothelial cells	7/60 (11%)	53/60 (88%)	Tumor endothelial cells	30/40 (75%)	10/40 (25%)	<0.0001

*Quantification of immunostaining for cysteine cathepsins (CTSL and CTSB) was performed by calculating the H score as described in the methods section. Statistical analysis of the results was carried out by Chi-Square Test. GB, gallbladder; GBC, gallbladder cancer*.

### CTSL and CTSB mRNA Expression in GBC

**Figure 5:**
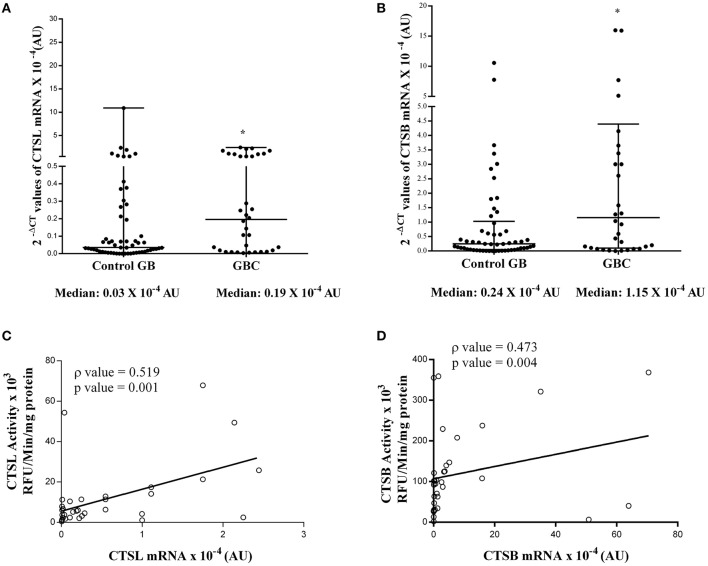
Expression of cysteine cathepsin mRNA in GBC. Total cellular RNA isolated from gallbladder tissues obtained from controls (*n* = 54) and GBC (*n* = 34) patients were reverse transcribed and subjected to qRT-PCR using specific primers for **(A)** CTSL and **(B)** CTSB. After normalization with housekeeping gene 18S, 2^−ΔCT^ values were plotted and represented in a dot plot. Relative mRNA levels of these cathepsins were significantly elevated in GBC as compared to controls. Spearman Rank Correlation analysis between **(C)** CTSL activity and mRNA **(D)** CTSB activity and mRNA expression in GBC patients revealed transcriptional upregulation of these proteases in this malignancy. All values presented in the median with interquartile range (IQR). Results were analyzed using the Mann Whitney *U* test; *p ≤ 0.050 was considered statistically significant.

The quantitative real-time PCR analysis was used to assess the mRNA expression of these cysteine cathepsins in both tumor and control gallbladder tissues. For this purpose, the total RNA was isolated from flash-frozen gallbladder tissue obtained from both GBC patients and controls. However, qPCR could be performed only in 34 GBC patient tissues and 54 controls. In the remaining cases, either the tissue sample was limited, or the quality of isolated RNA was not up to the mark. The median 2^−ΔCt^ values of CTSL mRNA in GBC patients was 0.19 × 10^−4^ AU (range 0.01–0.99) as compared to 0.03 × 10^−4^ AU (IQR 0.01–0.22) in controls. Thus, a significant increase in median CTSL mRNA levels was observed in GBC (*p* = 0.023, Figure 5A). A similar increase of around 5-fold in median 2^−ΔCt^ values of CTSB mRNA (1.15 × 10^−4^ AU, IQR 0.09–4.39) was also noticed in GBC patients with respect to the controls (0.24 × 10^−4^ AU, IQR 0.04–1.02; *p* = 0.015, Figure 5B).

To ascertain the degree of association (if any) between enzymatic activities and mRNA levels of these proteases, Spearman rank correlation analysis was performed. As evident from this analysis, we observed a positive correlation (ρ = 0.519, *p* = 0.001, Figure 5C) between CTSL enzyme activity and its mRNA levels. Similarly, a strong positive correlation between CTSB enzyme activity and its mRNA expression was also evident in GBC patients (ρ = 0.473, *p* = 0.004, Figure 5D). Therefore, these findings indicate that the transcriptional upregulation of these proteases in GBC may be responsible for the higher protein levels and increased enzymatic activities of both CTSL and CTSB in this malignancy.

### Serum Levels of CTSL and CTSB in GBC

**Figure 6:**
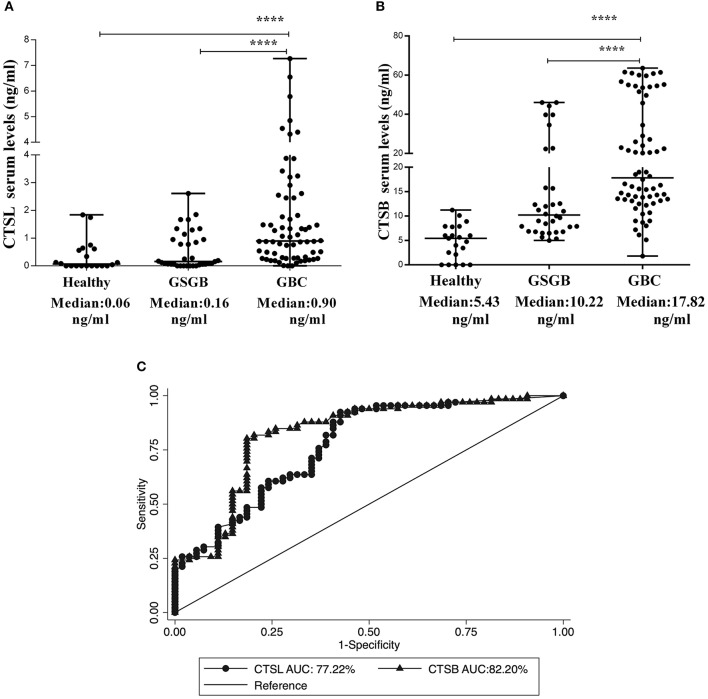
Elevation in serum levels of CTSL and CTSB in GBC patients. Dot plot representation of **(A)** CTSL and **(B)** CTSB and in the serum of healthy individuals (*n* = 20), GSGB (*n* = 34), and GBC patients (*n* = 66) as estimated by ELISA. Serum levels of CTSL and CTSB were significantly higher in GBC patients as compared to both healthy individuals and patients with gallstone disease. Median serum values have been represented by the horizontal bar in the middle. Results were analyzed by the Mann-Whitney *U* test, and values significantly different from both GS and healthy controls have been marked by ****p < 0.0001. **(C)** ROC curve analysis of serum cysteine cathepsins showing excellent diagnostic performance of both CTSL (AUC−77%) and CTSB (AUC −82%) in distinguishing GBC patients from control subjects.

Having established the diagnostic and prognostic significance of CTSL and CTSB overexpression in the tumor tissues of GBC, it was of interest to investigate if the levels of these cathepsins in serum could accurately discern GBC patients from controls. To test this hypothesis, ELISA was employed to estimate the expression of CTSL and CTSB in the serum of GBC patients and controls. The median CTSL value in GBC patients was found to be 0.90 ng/ml (IQR 0.28–2.45). This value was significantly higher as compared to the serum levels of CTSL in both GSGB (0.16 ng/ml, IQR 0.05–0.95) and healthy controls (0.06 ng/ml, IQR 0–0.60, *p* < 0.0001, Figure 6A). Similarly, the median CTSB antigenic levels in GBC were found to be elevated (17.8 ng/ml, IQR 13.3–46.7) with respect to its level in both GSGB (10.20 ng/ml, IQR 6.80–17.40) and healthy controls (5.43 ng/ml, IQR 1.07–7.8, *p* < 0.0001, Figure 6B).

Serum antigenic expression of CTSL and CTSB obtained from GBC patients and controls was used to assess the diagnostic potential of these molecules. The ROC-based assessment revealed an optimal cut-off value of 0.30 ng/ml for CTSL, with 72% diagnostic sensitivity and an AUC value of 77% in distinguishing GBC from controls (Figure 6C, Table 4). Correspondingly, for CTSB, the chosen cutoff value was 11.6 ng/ml, with an excellent diagnostic sensitivity of 83% and an AUC value of 82% (Figure 6C, Table 4). Hence, our results for the first time demonstrate the diagnostic significance of both CTSL and CTSB as novel blood-based biomarkers in GBC.

**Table 4:** Assessment of diagnostic performance of serum cysteine cathepsins in GBC.

**Cysteine cathepsins** **(Cut off values)**	**AUC** **(%, 95% CI)**	**Sensitivity** **(%, 95% CI)**	**Specificity** **(%, 95% CI)**	**PPV** **(%, 95% CI)**	**NPV** **(%, 95% CI)**
CTSL (0.30 ng/ml)	77 (68–85)	72 (60–82)	63 (48–75)	70 (58–80)	65 (50–77)
CTSB (11.60 ng/ml)	82 (74–90)	83 (71–90)	74 (60–84)	79 (67–88)	78 (64–88)

*AUC were calculated using receiver–operating characteristic (ROC) curves. To obtain the cut off values of cysteine cathepsins, the sum of sensitivity and specificity were maximized*.

## Discussion

GBC is an aggressive malignancy that has the highest rate of mortality out of all biliary tract cancers. Despite advancements in several diagnostic modalities, strategies for early detection of this cancer remain limited. The majority of gallbladder carcinomas cases are detected at a late stage due to the paucity of specific symptoms early in the disease (19). Thus, investigations into the identification of novel biomarkers and methods to identify asymptomatic diseases are urgently needed to improve the overall survival of patients with GBC.

Lysosomal cysteine cathepsins (CTSL and CTSB) are degradative enzymes playing a pivotal role in tumor invasion and metastasis. In the present study, we have demonstrated a significant increase in the serum levels of CTSL and CTSB in GBC patients as compared to their levels in chronic cholecystitis and healthy controls. Furthermore, secreted levels of these cathepsins may serve as robust diagnostic markers, with an AUC of 77% and a sensitivity of 72% for CTSL and an AUC value of 82% with a sensitivity of 83% for CTSB, in distinguishing GBC patients from both chronic cholecystitis with benign gallbladder disease and healthy individuals.

Another interesting finding of our study is that spectrofluorometric based estimation in surgically resected gallbladder tissues revealed significantly higher enzymatic activities of cysteine cathepsins in GBC as compared to controls. Furthermore, the activity of CTSL+B and CTSB showed a significant association with tumor stage and lymph node involvement in GBC patients. Mirroring our findings, Lah et al. (20) also demonstrated higher activity and protein expression of both CTSL and CTSB in invasive breast carcinoma tissues as compared to the controls. It is noteworthy that the overexpression of CTSL and CTSB has also been implicated in the progression of colorectal cancer (21). Previously, our lab has established the role of CTSL and CTSB as a prognosticators of poor outcome in pediatric AML patients (22). The findings in our current study further demonstrate the prognostic significance of cysteine cathepsins in GBC. Results of Kaplan Meier survival analysis revealed reduced overall survival in GBC patients with higher combined CTSL+B activity, indicating the clinical utility of these cathepsins as prognostic markers of disease severity.

Higher levels and altered localization of cysteine cathepsins, including CTSL and CTSB within the tumor microenvironment, are essential for tumor growth, invasion, and neovascularization (23). In the present study, we employed immunohistochemical analysis to assess the expression and localization of CTSL and CTSB in GBC tissues and observed a remarkable increase in the expression of both these proteases in histologically proven GBC tissues as compared to control gallbladder tissues with chronic cholecystitis. The immunostaining pattern of these cathepsins revealed prominent expression and localization in tumor cells, tumor endothelial cells, and tumor-associated macrophages in GBC, whereas weak cytoplasmic expression was noted mainly in epithelial and endothelial cells in controls. Our data give credence to the previous finding, which demonstrated the prominent expression of CTSL and CTSB in tumor cells as well as in vascular endothelial cells of glioma (24). Our results are also consistent with the reports of Vasiljeva et al. (25) who observed an association between tumor macrophage-specific expression of CTSB with an increased incidence of lung metastasis in the mice model of breast carcinoma. Interestingly using iTRAQ based quantitative proteomics, Sahasrabuddhe et al. demonstrated over-expression of two other lysosomal cysteine proteases, namely cathepsin H and cathepsin Z in GBC (15). Thus, the results of the present study taken together with the findings of Sahasrabuddhe et al. suggest that overexpression of CTSL and CTSB in the tumor microenvironment of GBC could be a contributing factor in promoting disease aggressiveness.

Our lab has previously delineated the role of transcriptional as well as post-transcriptional up-regulation of CTSL expression in several cancers (26–30). Therefore, to outline the mechanism and further corroborate the observed increase in expression/activity of these cathepsins in GBC, mRNA levels of CTSL and CTSB were assessed in GBC patients by quantitative real-time PCR. Our results revealed a significant increase in the mRNA levels of both CTSL and CTSB in GBC as compared to controls. The increased mRNA expression exhibited a strong positive correlation with enzyme activities of both these proteases in tumor tissues, thereby suggesting the role of increased mRNA levels in elevating the expression of CTSL and CTSB. To the best of our knowledge, this is the first clinical study that not only establishes the importance of cysteine cathepsins (CTSL and CTSB) in the pathogenesis of GBC, but also highlights the need for future research to further understand the molecular mechanism of CTSL and CTSB over expression in this carcinoma.

In summary, we have demonstrated the upregulation of CTSL and CTSB expression and their clinical association with aggressiveness and poor survival in GBC patients. Furthermore, elevated serum levels of CTSL and CTSB were able to distinguish patients with active disease from controls with excellent diagnostic accuracy in our cohort. Our study establishes the need for further investigation into the use of CTSL and CTSB as screening tools in larger patient cohorts of GBC.

## Data Availability Statement

All datasets generated for this study are included in the article/Supplementary Material.

## Ethics Statement

Ethical clearance from the Human Ethics Committee of All India Institute of Medical Sciences was obtained before commencement of the study (IESC T-244/2012), and informed consent from all patients or their legally acceptable representatives was taken before their inclusion.

## Author Contributions

SM: performed all the experiments, collected samples, analyzed data, and drafted the manuscript. RP, ND, and PS: planning the study, interpretation of clinical results, and manuscript preparation. BT: statistical data analysis. MK and RS: helped in experiments as well as in writing and correction of the manuscript. RY: examined, reviewed, and scored the immunohistochemistry. SC: conceptualized the project, planned experiments, obtained funding for the project, provided infrastructure and resources for the work, analyzed and interpreted the results and corrected and finalized the manuscript. All authors reviewed and approved the final draft submitted.

### Conflict of Interest

The authors declare that the research was conducted in the absence of any commercial or financial relationships that could be construed as a potential conflict of interest.
