# Supplementary material for: Expression and Clinical Implications of Cysteine Cathepsins in Gallbladder Carcinoma
Source: Front Oncol. 2019 Nov 22;9:1239. doi: 10.3389/fonc.2019.01239 (PMC6883407; doi:10.3389/fonc.2019.01239)
Supplement: Supplementary file 1 [file Table_1.DOC]

**Supplementary Table 1. List of Primers used for Quantitative Realtime PCR**

| **Target gene** | **Name** | **Oligonucleotides Sequence** | **Amplicon Size** |
| --- | --- | --- | --- |
| **Cathepsin L** | CTSL-RTF | 5’-GACTCTGAGGAATCCTATCCA-3’ | 180bp |
|  | CTSL-RTR | 5’-AAGGACTCATGACCTGCATCAA-3’ |  |
| **Cathepsin B** | CTSB-RTF | 5’-TGTAATGGTGGCTATCCTGCT-3’ | 180bp |
|  | CTSB-RTR | 5’-AGGCTCACAGATCTTGCTACA-3’ |  |
| **18S** | 18S-RTF | 5’-GTAACCCGTTGAACCCCATT-3’ | 180bp |
|  | 18S-RTR | 5’-CCATCCAATCGGTAGTAGCG-3.’ |  |
